# The societal cost of treatment-seeking patients with borderline personality disorder in Germany

**DOI:** 10.1007/s00406-021-01332-1

**Published:** 2021-10-04

**Authors:** Till Wagner, Nele Assmann, Sandra Köhne, Anja Schaich, Daniel Alvarez-Fischer, Stefan Borgwardt, Arnoud Arntz, Ulrich Schweiger, Eva Fassbinder

**Affiliations:** 1 Department of Psychiatry and Psychotherapy, University of Lübeck, Ratzeburger Allee 160, 23538 Lübeck, Germany; 2 Institute of Neurogenetics, University of Lübeck, Lübeck, Germany; 3 Institute of Systems Motor Science, University of Lübeck, Lübeck, Germany; 4 Department of Clinical Psychology, University of Amsterdam, Amsterdam, The Netherlands; 5 Department of Psychiatry and Psychotherapy, Christian-Albrechts-University Kiel, Kiel, Germany

**Keywords:** Borderline personality disorder, Health economics, Cost-of-illness, Psychiatric care

## Abstract

According to previous research, borderline personality disorder (BPD) is associated with high cost-of-illness. However, there is still a shortage of cost-of-illness-studies assessing costs from a broad societal perspective, including direct and indirect costs. Further, there are considerable differences in the results among the existing studies. In the present study, 167 German men and women seeking specialized outpatient treatment for BPD were included. We assessed societal cost-of-illness bottom-up through structured face-to-face interviews and encompassed a wide range of cost components. All costs were calculated for the 2015 price level. Cost-of-illness amounted to € 31,130 per patient and year preceding disorder-specific outpatient treatment. € 17,044 (54.8%) were direct costs that were mostly related to hospital treatment. Indirect costs amounted to € 14,086 (45.2%). Within indirect costs, costs related to work disability were the most crucial cost driver. The present study underlines the tremendous economic burden of BPD. According to the present study, both the direct and indirect costs are of significant importance for the societal costs associated with BPD. Besides the need for more disorder-specific treatment facilities for men and women with BPD, we assume that education and employment are topics that should be specifically targeted and individually supported at an early stage of treatment.

Trial Registration: German Clinical Trial Registration, DRKS00011534, Date of Registration: 11/01/2017, retrospectively registered.

## Introduction

Borderline personality disorder (BPD) is a severe and prevalent mental disorder [1]. According to current classification systems [2, 3], BPD is characterized by a pervasive pattern of instability of interpersonal relationships, self-perception, and emotion regulation, as well as marked impulsivity. Due to recurrent emotional crises, men and women with BPD use a wide range of mental healthcare services, including frequent emergency room visits, hospital admissions, and medication [4–6]. Furthermore, BPD is strongly associated with other mental disorders [7] and severe medical diseases [8, 9]. Hence, they also frequently visit physicians for medical reasons [10]. Moreover, men and women with BPD are significantly impaired in psychosocial and occupational functioning, with a high proportion classified as severely disabled according to German law, unemployed, and relying on social welfare [11, 12]. In addition, family members are considerably burdened as they are often involved in interpersonal problems of men and women with BPD and their emotional crisis [13, 14]. Accordingly, BPD is associated with significant impairments in quality of life [15] and high cost-of-illness [16–20].

However, BPD-related costs vary considerably among existing studies ranging from € 9174 per patient and year in a German study [21] and € 56,731 per year and patient in a British study [22].^1^ Differences in results are mainly related to different recruitment settings and patient samples, differences in national healthcare systems, and methodological differences in assessing costs.

First, cost-of-illness analysis can be conducted from several different perspectives (e.g., health insurance funds, government, society), and the perspective determines which costs are included in the analysis. The most comprehensive perspective is the societal perspective taking into account all costs irrespectively of who bears them. At the same time, the cost analysis conducted from the same perspective can still considerably differ in the number of cost components included in the study.

Second, there are different methodological approaches to obtain cost data [25]. In *bottom-up studies*, costs are assessed on the patient level based on self-reports or individualized claims data of payers such as health insurance providers. In *top–down studies*, total costs per health care sector are derived from databases such as national registers and health reports and are assigned to a specific diagnosis. While top–down studies can rely on large samples, costs per patient must be calculated based on prevalence data or several assumptions since little disease-specific information is available in public databases.

In sum, BPD has substantial impacts on every area of life, and costs accrue far beyond the health care sector. However, there is still a shortage of cost-of-illness-studies assessing BPD-related costs from a societal perspective.

In the present study, we aim to expand and further clarify the knowledge on the cost-of-illness of BPD. We assessed annual costs bottom-up from a societal perspective in a relatively large sample of 167 German men and women with BPD seeking specialized outpatient treatment. We included a wide range of direct costs (costs related to medical and non-medical resource consumption) and indirect costs (costs due to loss of productivity).

A clear picture of the economic burden of BPD is crucial to inform decisions on resource allocation in a budget-constrained healthcare system and to develop and implement specific strategies in BPD treatment leading to both a higher quality of life for patients but also a reduction of costs for society.

## Materials and methods

### Setting and recruitment

The present cost-of-illness study was conducted as part of the PROgrams for Borderline Personality Disorder study (Pro*BPD), a randomized controlled trial comparing the clinical effectiveness and cost-effectiveness of outpatient Dialectical Behaviour Therapy (DBT) and Schema Therapy (ST) [26] (German Clinical Trial Register: DRKS00011534). Within the Pro*BPD study, patients are offered a treatment program for 1.5 years with either DBT or ST. The PRO*BPD study is conducted at the Department of Psychiatry and Psychotherapy, University of Lübeck, Germany, that provides inpatient and outpatient services for all major psychiatric disorders. Patients were referred to the study by the different units within the Department of Psychiatry and Psychotherapy (inpatient and outpatient services) and other mental healthcare providers, including local private psychologists. Potential participants were informed about the study and invited for a screening to check the inclusion and exclusion criteria. Data for the present cost-of-illness study were collected from March 2015 to January 2019.

### Participants

Patients aged between 18 and 65 years were included in the Pro*BPD trial and thus into the present cost-of-illness analyses if (i) they had a primary diagnosis of BPD according to DSM-IV—diagnosed with the Structured Clinical Interview for DSM-IV for Axis II (SCID-II)—, (ii) had a severity score higher than 20 points on the Borderline Personality Disorder Severity Index Version 4 (BPDSI) [27], (iii) indicated that they were committed to participate in the treatment program for 1.5 years, plan to attend the therapy sessions within the treatment program regularly, and denied relocation plans. In addition, they had to provide written informed consent after obtaining written information and a complete oral description of the study. For the purpose of high ecological validity, only a few exclusion criteria were implemented. Exclusion criteria were a major psychotic disorder (lifetime diagnosis), IQ below 85, and an acute severe substance dependence requiring detoxification treatment. The latter patients could participate in the study after detoxification, 4 weeks of abstinence, and if they were willing to further work on abstinence during outpatient treatment. In sum, baseline cost data were collected from 167 patients. Of these, 162 patients participated in the treatment program of the Pro*BPD study. Five more patients completed the diagnostic procedure, including the baseline cost assessment but did not start treatment after randomization (non-starter).

### Data collection: assessment of resource consumption and productivity loss

Resource consumption and productivity loss were assessed from a societal perspective. We used patient-level data and assessed resource consumption and productivity loss through structured face-to-face interviews (“Cost Interview”) covering the previous 12 months.

The Cost Interview used in the current study was validated afore and used in a previous study on costs of BPD [19]. Before the current study, modifications were made to improve clearness/comprehensibility, and additional items were incorporated into the Cost Interview. Items related to direct medical costs included psychiatric and general hospital days, days in a psychiatric day clinic, drug intake, visits to emergency rooms, outpatient psychotherapists, psychiatrists, general practitioners, medical specialists, occupational therapists, physical therapists, community-based counselors, and crisis centers. Items related to direct non-medical costs included assisted living programs, informal care, which occurs when significant others take over domestic tasks without payment. We also assessed incidents of delinquent behavior (e.g., traffic offenses, bodily injury) and their associated consequences, such as police operations or monetary compensation for damages. Productivity loss was captured with questions on employment status, the source of income, gross income, the number of weeks of employment, unemployment, and work disability during the previous 12 months. Employed patients were asked about their average weekly work hours and the number of days they were absent from work due to illness.

It is essential to mention that the assessment of resource consumption and productivity loss was exclusively based on patients’ self-reports. This also applied to direct non-medical resource consumption. For example, in the case of informal care, we asked patients to what extent relatives helped them with household chores. Similarly, in the case of traffic offenses, patients estimated the amount of the corresponding fines. Information on resource consumption and productivity loss was collected in a two-stage process: Patients received the Cost-Interview before the interview appointment for an initial self-evaluation. In the interview appointment, missing items were assessed by the interviewer. In the rare cases when information was missing, the patient was called again.

During the interview appointment, it was also determined whether resource consumption and productivity loss were due to mental disorders or medical diseases. In the current study, we only distinguished between the costs due to psychological disorders and somatic diseases. Wagner et al. [19] found that it was difficult to clearly distinguish between BPD-related costs and costs due to other mental disorders because some symptoms (e.g., binge eating) are both a diagnostic criterion of BPD and other mental illnesses.

### Cost calculation

To calculate direct costs, we multiplied resources by their corresponding unit cost (i.e., the cost of a particular medical or non-medical treatment). Unfortunately, there is no obligatory unit cost list in Germany covering all medical and non-medical services used by men and women with BPD. At the same time, standardized unit costs for many areas have been calculated for Germany from the societal perspective and recently been published.

For medical services such as hospital treatments and outpatient medical care, we referred to the unit costs calculated by Bock et al. [28]. For community-based treatments, we used the unit costs calculated by Grupp et al. [29].

Since the above publications do not include unit costs for the areas of psychotherapy and medication, we calculated the corresponding costs from the perspective of the payers. Accordingly, unit costs for individual and group psychotherapy were calculated based on the current physician's fee schedule for psychotherapy [30].To calculate costs related to drug intake, for each psychotropic drug listed in the cost interview, we multiplied the number of packages by the pharmacy sales price listed in the “Rote Liste®” [31].

Unit costs related to delinquent behavior were dependent on the consequences of a specific behavior. In the case of property damage, thievery, and traffic offenses, unit costs were based on the patient's information about the value of the damaged item or the fine. Treatment costs related to bodily injury were calculated in the above-mentioned medical unit costs. If the police or an emergency ambulance was involved in an incident, we also added mean costs for a police operation or emergency ambulance operation. Unit costs for a police operation were calculated based on the assumption that two police officers are involved in an operation and that an operation takes one hour and based on the relevant mean labor costs for police officers [32]. Unit costs for an emergency ambulance operation were based on an interview with a health insurance company in Lübeck (VIACTIV, personal communication on 18th of June 2018).

All unit costs were adjusted for the 2015 price level with the consumer price index [23] since data collection started in 2015. Table 1 presents the value of the corresponding unit cost for medical and non-medical services.

**Table 1:** Value of the unit cost for each medical and non-medical service

Resource consumption	Unit	Unit cost (in €)
Direct medical costs		
Hospital		
Psychiatric hospital	Day	360
Psychiatric day program	Day	234
General hospital	Day	638
Intensive care unit	Day	1442
Outpatient medical care		
General practitioner	Visit	21
Psychiatrist	Visit	48
Medical specialist	Visit	20–65
Psychotropic drugs	Per pack	Various
Physical therapy	Visit	17
Occupational therapy	Visit	40
Outpatient psychotherapy		
Individual therapy	Visit	97
Group therapy	Visit	47
Emergency care		
Emergency room	Visit	46
Emergency medical service	Visit	31
Emergency ambulance	Incident	523
Community-based treatments		
Psychological counseling	Visit	104
Counseling center, other	Visit	104
Crisis service (non-medical)	Visit	25
Social psychiatric service	Visit	117
Individual case aid/family assistance	Hour	31
Direct non-medical costs		
Assisted living (outpatient)	Day	23
Assisted living (inpatient)	Day	83
Informal care	Hour	14
Consequences of delinquent behavior		
Police operation	Incident	95
Property damage	Incident	Property value
Bodily harm	Incident	Treatment cost injured person
Traffic offense	Incident	Monetary fine
Thievery	Incident	Cost stolen item

All unit costs were adjusted for the year 2015 price level and rounded to whole decimal places

The calculation of indirect costs was initially carried out in the primary analysis with the Human Capital Approach and in a sensitivity analysis with the Friction Cost Approach [33].

According to the Human Capital Approach, we counted productivity loss due to days of incapacity to work and productivity loss due to work disability. To calculate costs related to incapacity to work, we considered those employees engaged in an employment relationship that carries obligatory social insurance. We multiplied days of incapacity to work by the individual labor costs for each patient with a paid labor, including the individual gross wage plus non-wage labor costs [34]. Work disability-related costs were calculated based on a scenario analysis. Accordingly, we did not consider the number of social benefits the work disabled received but assumed that work disabled patients without their disorder would pursue a paid job and contribute to society's productivity. Here, we multiplied average monthly labor costs based on local average gender-adjusted gross wages weighted for full-time and part-time employment [35] plus average non-wage labor costs of 28% by the number of months the patients were work-disabled. Thereby, men's productivity loss was estimated based on an average labor cost of € 4307 per month and for women based on € 3105 per month, respectively.

According to the Friction Cost Approach, only the costs due to incapacity to work were considered calculating indirect costs. Here, 80% of individual labor costs were included in the calculation, and a friction period of 49 working days or 72 calendar days was assumed [36]. The friction period is defined as the time it takes to hire and instruct a new employee and restore the initial production level.

### Data entry and statistical analyses

Data entry and calculation of costs were performed with IBM SPSS statistic version 26. We used a non-parametric bootstrap resampling method with 1000 replications as an alternative to the geometric means as the cost data were skewed and not normally distributed. All costs are expressed in Euros with the price level of the year 2015.

## Results

The socio-demographic and clinical characteristics of participants are shown in Table 2.

**Table 2:** Sample socio-demographic and clinical characteristics (*N* = 167)

Variable	*M*	SD
Age in years	33.7	10.6

	*N*	%
Gender		
Female	132	79.0
Male	35	21.0
School education		
Higher education entrance qualification (A-levels, grade 12/13, German ‘Abitur’)	51	30.6
Intermediate school-leaving certificate (grade 10, German ‘Realschulabschluss’)	67	40.1
Basic secondary school (grade 9, German ‘Hauptschulabschluss’)	43	25.7
No graduation	6	3.6
Further education		
Study completed	13	7.8
Training completed	103	61.7
No training/study completed	51	30.5
Employment status		
Employed	22	13.2
Employed, currently on sick leave	8	4.8
Unemployed	48	28.7
Work disabled	53	31.7
Sheltered employment	5	3.0
Student	6	3.6
University student	6	3.6
Trainee	10	6.0
Homemaker	9	5.4

	*M*	SD
BPDSI^a^, total score	33.9	8.0
Number of SCID-II BPD criteria	7.2	1.3
Number of comorbid Axis-I-disorders	4.0	1.9
Number of comorbid Axis-II-disorders	1.4	1.1

(%) relates to the percentage of participants to whom the item was applicable

*M* mean, *SD* standard deviation

^a^Clinical characteristics of the sample were available for *N* = 164 patients due to *N* = 3 missings

### Total costs, total direct and indirect costs

Table 3 shows mean annual total costs, mean annual direct medical and direct non-medical costs, and mean annual indirect costs according to the Human Capital Approach. All costs are related to psychological problems.

**Table 3:** Mean annual total costs, mean annual direct medical and direct non-medical costs, and mean annual indirect costs according to the Human Capital Approach

	*M*	SD	Confidence intervals of the means^a^	Mdn	R
Total costs	31,130	28,630	26,975–35,360	22,481	333–104,477
Direct medical costs	15,321	16,829	12,974–17,715	9660	312–67,810
Direct non-medical costs	1723	5218	1024–2557	0	0–30,293
Indirect costs	14,086	18,972	11,314–16,922	0	0–51,686

All costs are related to psychological problems and are declared in €

All costs were adjusted for the year 2015 price level and rounded to whole decimal places

*M* mean, *SD* standard deviation, *Mdn* Median, *R* Range

^a^The 95% confidence intervals were calculated based on the bootstrapping method with 1000 replications

Of the total mean annual cost-of-illness, 49.2% were direct medical costs, 5.5% were direct non-medical costs, and 45.3% were indirect costs.

### Direct costs

Table 4 presents for each direct medical and direct non-medical cost component mean annual resource consumption and corresponding costs related to psychological problems. Besides, the percentage of each direct cost component on total direct costs is shown.

**Table 4:** Overview on direct costs

	Quantity		Unit of quantity	Costs (in €)		Confidence intervals of the means^a^	% on total direct costs
	*M*	SD		*M*	SD		
Direct medical costs							
Inpatient treatment	23.6	42.2	Days	8641^b^	15,378	6328–11,121	50.7
Psychiatric day program	16.1	30.1	Days	3774	7048	2722–4873	22.1
General practitioner	3.4	6.4	Visits	72	136	52–95	0.4
Medical specialists	0.5	1.4	Visits	21	65	12–31	0.1
Outpatient psychiatrist	4.2	5.6	Visits	188	264	152–228	1.1
Individual psychotherapy	11.2	9.4	Visits	1089	917	959–1235	6.4
Group psychotherapy	3.1	9.2	Visits	143	428	87–215	0.8
Psychotropic drugs	2.1^c^	1.7	Drugs	544	767	427–659	3.2
Emergency care	0.9	2.7	Visits	135	575	68–233	0.8
Physical and occupational therapy	3.1	11.8	Visits	123	469	60–200	0.7
Counseling and crisis center	1.8	8.2	Visits	141	396	87–202	0.8
Individual case aid/family assistance	24.5	148.4	Hours	450	1641	219–725	2.6
Direct non-medical costs							
Assisted living	12.9	59.3	Days	902	4416	315–1648	5.3
Informal care	49.0	210.8	Hours	698	3004	313–1223	4.1
Delinquent behavior	2.4	8.0	Incidence	123	513	59–209	0.7

Mean annual resource consumption and corresponding costs related to psychological problems and percentage of each direct cost component on total direct costs

All costs were adjusted for the year 2015 price level and rounded to whole decimal places

*M* mean, *SD* standard deviation, *Mdn* median

^a^The 95% confidence intervals were calculated based on the bootstrapping method with 1000 replications

^b^Costs stem from inpatient treatments in a psychiatric unit and inpatient treatment in a somatic unit due to psychiatric reasons such as self-mutilation

^c^While costs due to psychotropic drugs were calculated based on the number of packages of the drugs prescribed. Quantity here refers to the number of different kinds of psychotropic drugs taken

Patients used a wide range of direct medical and direct non-medical resources. By far, the most critical cost driver was costs associated with hospital treatment, which almost accounted for three-quarters of total direct costs. Ninety-four patients (56.3%) were at least once treated in hospital due to psychiatric reasons in the year before the Pro*BPD trial started. Among these patients, mean annual hospital days were 70.5 (SD = 46.1) days, and mean annual costs amounted to € 22,058 (SD = € 16,198).

In contrast, costs due to outpatient psychotherapy only made up 7.2% of total direct costs. The majority of patients (86.2%) used individual outpatient psychotherapy mainly in low frequency in the observation period. Within this subgroup, patients received an average of 13.0 (SD = 8.9) sessions per year.

Psychotropic drugs were taken by 133 patients (79.6%), which, on average, took 2.6 (SD = 1.5) psychotropic drugs.

Most patients (97.6%) visited a physician during the investigation period. When considering both visits due to psychological and somatic reasons, the total mean annual number of visits to physicians increased to 19.3 (SD = 19.8).

In the area of direct non-medical costs, resources were used by a small proportion of patients. Regarding informal care, 34 patients (20.4%) received help from relatives and friends due to psychological problems. On average, they received 240.7 h (SD = 419.5) of informal care amounting to mean annual costs of € 3430 (€ 5978).

In addition, 54 patients (32.3%) were involved in delinquent behavior in the investigation period, with, on average, 7.4 (SD = 12.8) incidents and related costs of € 381 (SD = € 851). Thereof, 54.5% of all incidents were attributable to property damage, 29.8% to traffic offenses, and 15.6% to police operations, thefts, and bodily injury.

### Indirect costs

In Table 5, based on the Human Capital Approach, indirect cost categories (incapacity to work and work disability) are presented.

**Table 5:** Overview on indirect costs

Indirect costs	Quantity		Unit of quantity	Costs (in €)		Confidence intervals of the means^a^	% on total indirect costs
	*M*	SD		*M*	SD		
Incapacity to work	14.8	62.7	Days	1484	6672	574–2597	10.5
Work disability	3.8	5.3	Months	12,602	18,789	9838–15,574	89.5

Mean annual days of incapacity to work and mean annual months in work disability and corresponding costs related to psychological problems based on the Human Capital Approach and percentage of each indirect cost component on total indirect costs

All costs were adjusted for the year 2015 price level and rounded to whole decimal places

*M* mean, *SD* standard deviation

^a^The 95% confidence intervals were calculated based on the bootstrapping method with 1000 replications

Within indirect costs, costs related to work disability showed to be the most significant cost driver. Fifty-three patients (31.7%) were work-disabled throughout the year due to a combination of BPD symptoms and chronic somatic problems.

Costs related to incapacity to work were related to 36 patients (21.6%) in employment at any time during the investigation. On average, those patients had a gross monthly earning of € 2094 (SD = € 977). Mean annual sick days were 75.3 days (SD = 119.5) in this patient subgroup. Of those, 68.5 days (SD = 121.9) were due to psychological problems, and the corresponding mean annual costs amounted to € 6882 (SD = € 13,151) within the subgroup of working patients.

According to the Friction Cost Approach, costs due to incapacity to work, and thus total indirect costs, amounted to € 436 (SD = € 1508) per patient and year across the entire sample. The corresponding mean annual days of incapacity to work related to psychological problems were 5.5 (SD = 17.5). The significantly lower costs due to incapacity to work compared to the Human Capital Approach were related to the fact that the number of days of incapacity to work exceeded the friction period of 49 days for seven patients.

## Discussion

### Main findings

In the present study, we assessed societal cost-of-illness in a sample of 167 German men and women with BPD seeking specialized outpatient treatment and included a wide range of cost components. Cost-of-illness amounted to € 31,130 per patient in the year preceding disorder-specific outpatient treatment. Thereof, € 17,054 (54.7%) were direct costs, while indirect costs amounted to € 14,085 (45.3%). Several significant findings emerge from our study.

First, our study underlines the tremendous economic burden of BPD. Mean annual BPD-related costs calculated in the present study exceed those in patients with many other mental diseases by far. By comparison, societal cost-of-illness calculated bottom-up in a German sample of patients with depression recruited in a primary care setting was € 7139 per patient and year [37]. Thereof, 59% (€ 4197) was attributable to indirect costs. In contrast to the present study, direct non-medical costs were not included in this study. For German patients with alcohol dependence, the mean annual costs calculated bottom-up from a societal perspective were € 11,130 [38]. Almost 70% (€ 7669) of total costs were indirect costs. However, comparisons between different cost-of-illness studies should be treated with caution due to differences in recruitment settings, the assessment of costs, and a wide range of cost components included in the cost calculations.

Second, the costs found in the present study exceed the costs calculated in the majority of previous studies in men and women with BPD. The two German cost studies by Wunsch et al. [20] and Bode et al. [21] both used top–down approaches and calculated costs from the perspective of the health insurance funds. Here, cost estimations were based on larger samples, but a significantly smaller range of cost components was included in the cost calculations. In the study of Wunsch et al. mean annual cost-of-illness amounted to € 16,954, and Bode et al. calculated a total cost-of-illness of € 9174 per patient and year. Salvador Carulla et al. [17] also used a top–down approach but assessed costs from a societal perspective. Here, the societal cost-of-illness of Spanish men and women with BPD was € 13,609 per patient and year. In contrast, societal cost-of-illness amounted to € 40,441 in a nationwide Danish study [16]. In this study, costs were calculated based on individualized claims data, and psychiatric and somatic costs were considered. In estimating indirect costs, the authors also included transfer payments such as unemployment benefits. Furthermore, in British men and women with BPD seeking specialized partial hospital treatment, mean annual direct medical costs even amounted to € 56,731 [22].

From a methodological point of view, the approach chosen in our study is comparable to those in the cost studies by Wagner et al. [19] and van Asselt et al. [18]. As in our study, both studies targeted patients seeking specialized outpatient treatment, and costs were assessed bottom-up from a societal perspective.

Wagner et al. found a total cost-of-illness of € 30,220 per patient and year in a sample of 47 German men and women with BPD. Thereof, 67.9% (€ 20,519) were direct costs, and 32.1% (€ 9701) were indirect costs. In the study by van Asselt et al., the total cost-of-illness amounted to € 22,849 per patient and year in a sample of 88 Dutch patients. Here, only 21.7% (€ 4958) of total costs were direct healthcare costs, while 26.5% (€ 6055) were direct non-medical costs and more than 50% (€ 11,836) of total costs were indirect costs.

The third significant finding of our study is that hospital treatment costs were by far the most crucial cost driver, accounting for almost 75% of direct costs. Patients in our study were, on average, 39.7 days in the hospital due to BPD in the year preceding the assessment. This finding is in line with the other German cost-of-illness-study [19] and underlines that psychiatric hospital treatment plays a crucial role in the care of men and women with BPD in Germany. In contrast, mean annual BPD-related hospital days were 16 days in Dutch BPD-patients [18] and 24 days in Spanish BPD-patients [17].

At the same time, compared to the German study by Wagner et al., mean annual hospital days were lower by almost 12 days in the present study. This result initially is surprising since we included severely ill men and women with BPD and many comorbid problems. We assume that this finding is linked to specific characteristics of the treatment setting in Lübeck. The Department of Psychiatry and Psychotherapy has a long tradition and expertise in treating men and women with BPD. Treatment structures are tailored to the needs of patients with severe illness and aim at avoiding short-term inpatient admissions.

Our study underscores the great importance of indirect costs within societal costs in men and women with BPD as a last major finding. Within indirect costs, costs related to work disability were the most significant cost driver. Almost one-third of the sample was disabled for work, and almost 30% of the sample was unemployed, pointing to deficient occupational functioning of the patients investigated.

Costs related to work disability calculated in our study (*M* = € 12,602) were higher compared to those calculated in the Dutch study by van Asselt et al. (*M* = € 9162) and the German study by Wagner et al. (*M* = € 7999). Similar to our study, 32% of the patients in the Dutch study were fully disabled, and further, 9% were partially disabled. Higher costs related to work disability calculated in the present study are primarily related to the fact that a higher proportion of our sample was male than in the Dutch study (21% vs. 8%). This is because the calculation of work disability-related costs is based on gender-adjusted average gross income, and men, on average, have a higher income. In contrast, in the study by Wagner et al., only 18% of the sample was work-disabled, resulting in lower work-disability-related costs than in the present study.

Only a comparatively small proportion of indirect costs was related to days of incapacity to work. This finding is due to the small proportion of men and women with BPD employed in the regular labor market. Still, the economic relevance of work absenteeism in patients with BPD should not be underestimated. Mean annual days of incapacity to work of about 75 days calculated in our subsample of employed patients by far exceeded corresponding mean annual days of sick leave of 17.9 days in the German general population [39]. It can be assumed that being absent from work often increases the risk of losing one's job and results in unemployment.

Furthermore, the significance attributed to indirect costs crucially depends on the type of calculation. Indirect costs calculated using the Friction Cost Approach were significantly lower than those calculated using the Human Capital Approach. Overall, there is still a great debate about how lost productivity should be assessed [33]. Whereas the Human Capital Approach considers the potential productivity loss regardless of the level of employment, the Friction Cost Method is based on the assumption that in economies without full employment, costs only accrue up until another person replaces an employee.

In sum, we find the Human Capital Approach to be more appropriate to quantify productivity loss in men and women with BPD. Given the small number of working patients and the sizable proportion of patients that are disabled for work or unemployed in our sample, we assume that the Friction Cost Method underestimates indirect costs and does not adequately reflect the economic burden. In addition, the Friction Cost Approach assumes that full productivity is restored to the workplace after a relatively short recruitment and on-the-job training period. However, we also see that the Human Capital Approach leads to an overestimation of indirect costs. There currently is a good employment situation in Germany but no absolute full employment, which means that people in the workplace are replaceable to a certain extent.

Within the framework of this discussion, we would finally like to point out that unemployment is undoubtedly also an economic burden that is not quantified in either approach. The unemployment rate of 28.7% in our sample is significantly higher than the regional unemployment rate of about 6% [40], and we assume that some of the unemployed patients would be available for the labor market without their illness and thus contribute to the gross national product. At the same time, it is difficult to differentiate whether unemployment is related to BPD or other reasons.

### Limitations

Several limitations of this study must be taken into consideration. First of all, the generalizability of our results might be limited by the specific characteristics of the recruitment setting. Our Department is highly specialized in treating BPD and offers a wide range of treatment options for this patient group. Therefore, treatment utilization of patients in the current study might differ from the care-seeking behavior of patients in rural areas lacking disorder-specific treatments.

Also, specific selection effects might have influenced the costs assessed in the present study. Notably, we only included men and women with BPD actively seeking treatment. This selection criterion might have led to an overestimation of costs since treatment-seeking patients might experience a high burden of distress during the time preceding psychotherapy and thus might utilize treatments to a greater extent than people with BPD in the general population. They might also display greater BPD severity and higher comorbidity and be more impaired in psychological functioning leading to higher costs. At the same time, we might have underestimated costs in patients not actively seeking or even rejecting psychotherapy, thus causing substantial costs in other fields such as hospital treatment or prison terms.

Furthermore, assessment of costs was solely based on patients' self-report and, thus, patients' memory. Given the long retrospective period of one year and considering the full range of costs we assessed, our data's validity might be decreased by patients' inaccurate memory. Simultaneously, comprehensiveness is an essential advantage of our study, and top-down-cost studies lack important cost components such as direct non-medical costs. In future studies, the quality of data collection could be improved by checking patients’ information on specific cost components such as inpatient treatments or days of incapacity to work with claims data from health insurance providers or in the case of informal care, relatives could be directly asked to what extent they helped patients with household duties. Furthermore, the survey period could be shortened to the last 6 months to extrapolate the resource consumption and productivity loss to the whole of the past year.

Compared to previous bottom-up-cost studies in BPD, which attempted to only count costs as BPD-related costs directly related to BPD pathology, we distinguished whether costs were due to psychological disorders or medical diseases. Thus, we also counted costs as BPD-related costs related to symptoms of comorbid psychiatric disorders such as the depressive symptom of listlessness. On the one hand, this might have led to an overestimation of BPD-related costs. However, on the other hand, BPD can be viewed as the primary disease with many problems resulting from this condition. Moreover, it is difficult to clearly distinguish between BPD-related costs and those costs related to other mental disorders since some symptoms (e.g., eating attacks, emptiness) are both part of the diagnostic criteria of BPD as well as of the diagnostic criteria of an Axis-1-disorder. Notably, the distinction between costs due to mental disorders and medical diseases might also have led to underestimating BPD-related costs in the present study as BPD is associated with physical health problems [7, 8, 41].

### Future directions in research

Future cost analyses should further examine the direct and indirect costs of men and women with BPD in different recruitment settings to gain an even more differentiated picture of the cost-of-illness of BPD. For example, it is desirable to examine the costs of patients seeking treatment in more rural areas and away from specialized treatment centers. Also, the costs of treatment-seeking patients should be compared to patients who do not seek treatment. In addition, there are several disorder-specific and clinically effective treatments for BPD [42] that are also associated with overall healthcare-cost offsets compared to treatment as usual [43, 44] and thus have tremendous economic potential. Future research should further investigate the long-term sustainability of evidence-based psychotherapies regarding the reduction of unspecific emergency treatments. Further, the success of specialized treatments for BPD will have to be gauged against how well they can promote reintegration in education and occupation. In future randomized controlled trials, the efficiency of evidence-based treatments for BPD should directly be compared to each other in cost-effectiveness and cost-utility analysis in terms of costs per quality-adjusted life-year (QALY) and costs per recovered patient providing an empirical basis for resource allocation decisions in health care policies. Here, we expect that our Pro*BPD trial [26] will help to reduce the existing research gap by investigating the efficiency of outpatient DBT and ST within the scope of a profound health economic evaluation.

### Clinical implications

The main objective of a cost-of-illness analysis is to determine the economic relevance of a disease and not primarily inform decision-making in health care. However, suggestions for better care of men and women with BPD can be derived from the results of this study. For example, the patients we studied used inpatient stays intensively, and most patients took at least one psychotropic drug, although drug treatment of BPD is not supported by the current evidence [45]. In contrast, outpatient psychotherapy was used only to a small extent. This is probably mainly because specialized outpatient treatment facilities for men and women with BPD are still limited [46, 47]. Thus, outpatient disorder-specific treatment services must be extended. Therefore, a recent challenge is to train more therapists in evidence-based psychotherapies for BPD and to motivate therapists in private practice that are already trained in evidence-based psychotherapies to treat those patients. In addition, better financial incentives should be offered for treating men and women with BPD as the German health care system does not stimulate outpatient treatment of complex mental disorders.

Furthermore, while pointing to deficient occupational functioning of the majority of the BPD-patients investigated, our study is in line with previous publications showing that the low psychosocial functioning of men and women with BPD remained stable over extended periods of time [48]. Accordingly, we assume that a more extensive range of services and approaches for men and women with BPD is strongly needed in occupational rehabilitation. Here, for certain patients, a paradigm shift might be promising, and we assume that employment should be targeted and individually supported at an early stage of treatment. While traditionally, in Germany, the first step in vocational rehabilitation is to treat and then engage people on the sheltered labor market, only a small percentage of patients can thus finally be integrated into the general labor market [49]. In contrast, supported employment programs such as individual placement and support (IPS) follow the strategy “place- and-treat-/train” instead of “train/treat-then-place” concurrently offering care and at the same time helping in finding employment [50, 51]. Thereby, specific barriers to employment, such as distance to the labor market and stigma, are dealt with.

Finally, in the field of prevention, early detection and treatment of BPD in youth and early adulthood and early interventions in high-risk families are of pivotal importance [52].

### Conclusions

In the present study, societal cost-of-illness was assessed comprehensively in a sample of men and women with BPD seeking specialized outpatient treatment in the clinical setting of a University psychiatric hospital. In conclusion, our study underlines the high social and economic relevance of BPD. Costs related to hospital treatment are the most crucial cost driver within direct costs in German patients. Furthermore, indirect costs are of significant importance within societal costs in patients. By now, the finding of the high cost-of-illness in men and women with BPD that are both due to high direct and indirect costs can be viewed as a robust finding across different recruitment settings, national social welfare systems, and despite methodological differences.

## Data Availability

The data sets used and analyzed during the current study are available from the corresponding author on reasonable request.

## Abbreviations

BPD: Borderline Personality Disorder
BPDSI: Borderline Personality Disorder Severity Index Version 4
DBT: Dialectical Behaviour Therapy
DSM-IV: Diagnostic and statistical manual of mental disorders, 4th edn
IPS: Individual Placement and Support
Pro*BPD: PROgrams for Borderline Personality Disorder study
QALY: Quality-Adjusted Life-Year
SCID-II: Structural Clinical Interview for DSM-IV for Axis II
ST: Schema Therapy

## Footnotes

1 In the following sections, to improve comparability among studies, all presented costs are adjusted for the 2015 price level with the consumer price index [23, 24] and converted into euros.
